# Perceptions of the value of traditional ecological knowledge to formal school curricula: opportunities and challenges from Malekula Island, Vanuatu

**DOI:** 10.1186/1746-4269-7-38

**Published:** 2011-11-23

**Authors:** Joe McCarter, Michael C Gavin

**Affiliations:** 1 School of Environmental Studies, Victoria University Wellington, PO Box 600, Wellington New Zealand

**Keywords:** Traditional ecological knowledge, formal education systems, contextualised education, cultural conservation, Vanuatu, Pacific islands

## Abstract

**Background:**

The integration of traditional ecological knowledge (TEK) into formal school curricula may be a key tool for the revitalisation of biocultural diversity, and has the potential to improve the delivery of educational objectives. This paper explores perceptions of the value of TEK to formal education curricula on Malekula Island, Vanuatu. We conducted 49 interviews with key stakeholders (local TEK experts, educators, and officials) regarding the use of the formal school system to transmit, maintain, and revitalise TEK. Interviews also gathered information on the areas where TEK might add value to school curricula and on the perceived barriers to maintaining and revitalising TEK via formal education programs.

**Results:**

Participants reported that TEK had eroded on Malekula, and identified the formal school system as a principal driver. Most interviewees believed that if an appropriate format could be developed, TEK could be included in the formal education system. Such an approach has potential to maintain customary knowledge and practice in the focus communities. Participants identified several specific domains of TEK for inclusion in school curricula, including ethnomedical knowledge, agricultural knowledge and practice, and the reinforcement of respect for traditional authority and values. However, interviewees also noted a number of practical and epistemological barriers to teaching TEK in school. These included the cultural diversity of Malekula, tensions between public and private forms of knowledge, and multiple values of TEK within the community.

**Conclusions:**

TEK has potential to add value to formal education systems in Vanuatu by contextualising the content and process of curricular delivery, and by facilitating character development and self-awareness in students. These benefits are congruent with UNESCO-mandated goals for curricular reform and provide a strong argument for the inclusion of TEK in formal school systems. Such approaches may also assist in the maintenance and revitalisation of at-risk systems of ethnobiological knowledge. However, we urge further research attention to the significant epistemological challenges inherent in including TEK in formal school, particularly as participants noted the potential for such approaches to have negative consequences.

## Background

The spread of western modes of formal education (defined here as institutionalised, chronologically graded, and hierarchically structured systems of education [1]) has been recognised as a key driver of global social change [2]. There are serious concerns, however, that formal education systems in some areas of the world do not adequately account for local knowledge and cultural diversity [3,4]. This results in school systems that are ineffective in attaining educational outcomes and which may actively erode cultural and linguistic diversity [5]. In consequence, there have been repeated calls over the last decade that local content should be included in education curricula [3,4,6]. In particular, the inclusion of traditional ecological knowledge (TEK)^i ^into formal schooling has been advocated by a variety of commentators [e.g. [7-9]].

Commentators have advanced four principal arguments for the inclusion of TEK in formal education systems. First, such approaches may contribute to the maintenance and revitalisation of TEK [7-9]. Recent quantitative work has shown that TEK can erode under the influence of various factors associated with modernisation, including integration into the cash economy [10-12] and linguistic erosion [12-14], as well as formal education [8,15,16]. Proponents have argued that the introduction of TEK at pre-school, primary, and secondary levels may increase rates of intergenerational knowledge transmission, in part by legitimising TEK for younger generations and giving it the same status as western knowledge [17]. This arrangement would leverage existing educational infrastructure, thus avoiding the need for costly novel interventions for TEK conservation [12]. In addition, interventions of this type would have the advantage of working through the predominant existing drivers of cultural change rather than against them, thus potentially mitigating the adverse effects of contemporary education models on TEK [9].

Second, a growing body of evidence suggests that the inclusion of TEK in formal school curricula might be an important means of increasing student's awareness and participation in environmental issues [18,19]. Most environmental knowledge is acquired at an early age (before 12) through sustained contact with the natural world [20], tutelage by parents [21,22], or play with peers [23-25]. Such experiences have a fundamental impact on individual cognition and ability to acquire local environmental knowledge and skills [25]. Formal education systems often remove children from this learning context at an early age, which can result in 'acquisition deprivation' and may inhibit a student's capacity to acquire environmental knowledge [18,23]. The use of TEK in school curricula may assist in mitigating impacts such as this.

Third, introducing local content such as TEK may contextualise formal education systems, making them more relevant and providing a better sense of place and identity to pupils [3-5,26]. This would address key criticisms of the classroom centred, exam based nature of western-style formal education, which can contrast with indigenous systems of cultural transmission [27]. Studies have noted that contextualised education systems that use locally relevant information can enhance understanding of curricular knowledge, in part through linking the theoretical knowledge of the classroom with practical, lived, reality [26,28]. Moreover, contextualised education systems can increase the acquisition of knowledge by empowering students, reinforcing learner self-esteem, and maintaining individual and collective cultural identity [28-30]. Similar approaches have received support at an institutional level, most notably from a keystone UNESCO document known as the Delors Report [31]. This report has formed the basis for curricular reform around the Asia-Pacific region [e.g. [32-35]], including the recently revised curriculum statement from the Vanuatu Ministry of Education [36].

Finally, the inclusion of TEK in school may provide a means of addressing the underlying power imbalance that often exists between centralised, state-run systems of education and minority or indigenous groups. Education is a key 'regime of power' through which a cultures conception of truth is maintained [37], and as such can play a critical role in the marginalisation of epistemological diversity [38]. In effect, this has meant that western-derived worldviews that promote values such as certainty, objectivity and instrumental rationality have dominated education systems and development rhetoric at the expense of local knowledge and practice [39]. In disregarding TEK and local content, formal education is argued to display 'systemic racism', and to foster separation between pupils and their community [40,41]. The inclusion of TEK in formal school, therefore, may mitigate this power differential and promote local participation and empowerment in education [6].

However, proposals to include TEK in formal education are controversial. Commentators have argued that western systems of formal education are, in fact, 'antithetical' to systems of indigenous knowledge [20,27], and have observed that the appropriation of TEK into school curricula may de-validate TEK by separating the knowledge from its cultural context [42]. In other discussions, scholars have argued for the universal application of western scientific education [43], or have contended that educational reform on the basis of 'cultural difference' may obscure the fluid nature of culture and impede progress toward educational outcomes [44].

In summary, academics and professionals from the fields of education, ethnobiology, and anthropology have called for the inclusion of TEK in formal education systems. However, such approaches may have negative consequences, which could lower the value of formal education or impact on the integrity of TEK. The integration of TEK and formal education will affect local TEK holders, parents, teachers, and education or cultural officials, all of whom are currently engaged in education and/or TEK conservation. The input of these stakeholders will be vital to the success of any such programme, but so far the literature does not include any examination of their perceptions.

The present study begins to fill this literature gap with a case study from Malekula Island, Vanuatu, by outlining the perceptions of ni-Vanuatu stakeholders on the value of TEK to formal education and *vice versa*. In this paper, we discuss the perceptions of interviewees related to three key questions: (i) Could TEK be legitimately included into the formal school system? (ii) How might this be achieved? (iii) What are the potential barriers to teaching TEK in schools?

## Methods

### Setting

**Figure 1:**
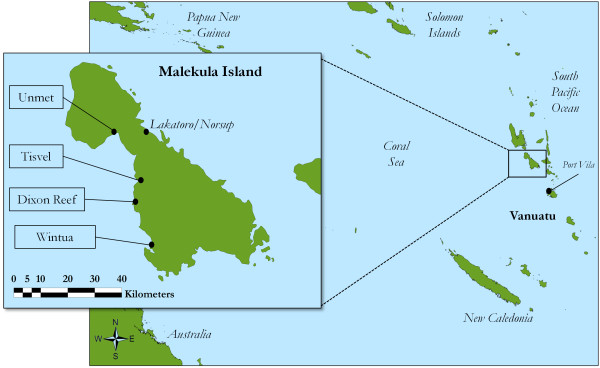
**Malekula Island and South Pacific, with focus communities**.

Malekula Island is the second-largest island in the Republic of Vanuatu (Figure 1). It is geographically diverse, with narrow coastal plains in the east and north, and rugged hills culminating in ranges of around 600-800 meters dominating the southern, western and interior sections [45]. Of the 206,756 ha of Malekula, approximately 75,000 ha is forested with native vegetation types including lowland rainforest, montane cloud forest, coastal vegetation, and secondary and cultivated woodlands [46]. Much of the remainder of the coastal plains have been converted to large commercial plantations of coconut (*Cocos nucifera*) and cacao (*Theobroma cacao*) [47,48]. Forest ecosystems on Malekula are not as diverse as those found elsewhere in the Asia-Pacific region [47]; however, Vanuatu is included as part of the East Melanesia biodiversity hotspot [49].

The population of around 27,000 are nearly all indigenous ni-Vanuatu [50], and speak at least 30 languages [51]. Per capita, Malekula may be the most linguistically diverse island in the most linguistically diverse country in the world [52]. Most Malekulans speak more than one vernacular language and are fluent in Bislama (a neo-Melanesian pidgin). Layered on top of this indigenous linguistic diversity are the colonial languages of English and French.

Malekulans fall into two broad cultural groups, with the V'ënen-Taut speaking Big Nambas inhabiting the north-west, and the linguistically diverse Small Nambas living in the remainder of the island. Inhabitants are rural and most rely on subsistence shifting cultivation systems of agriculture, based around the key staples of yam (*Dioscorea *spp.) and taro (*Colocasia *spp.), as well as near shore fishing and reef gleaning in coastal communities [53,54]. The administrative and trading centre of the island is Lakatoro/Norsup (population estimated at 335 in 1999 [55]), which is also the headquarters of the Malampa provincial office.

In Vanuatu TEK is an essential component of contemporary life. TEK has been documented in Vanuatu as a source of resilience to extreme weather events [56,57], as a facet of volcano hazard management [58], as a rich body of ethnomedical knowledge [59-63], and as a source of knowledge and practice for sustainable marine resource management [53]. More broadly, customary knowledge and practice (collectively termed *kastom *in Bislama^ii^) is described as the 'bedrock' of everyday life in Vanuatu, and there is a high level of cultural continuity throughout society [64]. Despite this, ni-Vanuatu scholars have expressed concern that the intergenerational transmission of TEK is at risk [e.g. [65]]. A number of factors are at play, including the priorities and disorganisation of central government, the policy and practice of religious organisations, and a lingering 'psychology of dependency' stemming from the colonisation experience [64-66]. To our knowledge TEK loss has not been empirically assessed in Vanuatu: however, erosion of TEK has been noted in similar circumstances elsewhere in Melanesia and Micronesia [e.g. [67,68]].

Vanuatu's formal education system has also been criticised for actively undermining traditional knowledge and *kastom*, on several fronts (see [69] for more detail on the following points). First, the curriculum excludes vernacular languages, despite regular debate on the issue.^iii ^Second, in maintaining a centralised curriculum based on New Zealand and Australian models and subjects (such as Science, English/French, and Maths), it ignores and devalues the diversity of indigenous knowledge and practice throughout the country. Third, schools usually employ non-local teachers and are not integrated with communities and traditional power structures. Fourth, in the community context, formal school is a large time commitment (at primary level around 30 hours a week, and at secondary level pupils often board away from their home community), which removes children from the traditional learning setting at an important age. As a result of these characteristics, commentators have claimed that the formal school system is not only ineffective in preparing pupils for employment in Vanuatu's urban centres, but that it also fails to teach the values and skills necessary for living more traditional, communal village lifestyles [36,64].

In response, the Vanuatu Cultural Centre (VCC)^iv ^runs a number of programs aimed at the maintenance and revitalisation of *kastom *and vernacular language. Two of these are of note here: the fieldworker program, which has been supporting volunteer local researchers to record their culture and language for over 30 years [70]; and a recent program that facilitates the teaching of TEK in the science curriculum by producing curriculum units for years 1 to 10 [71,72].

### Focus communities

Research was conducted in four rural communities on the West coast of Malekula Island: Unmet, Tisvel, Dixon Reef, and Wintua. Communities were selected in consultation with the VCC as locations where customary knowledge and practice form a crucial component of everyday life. Three of the four communities (with the exception of Tisvel) have primary schools, and Wintua and Unmet have secondary schools. Dixon Reef and Unmet are Francophone and Catholic, whereas Wintua and Tisvel are Anglophone and Presbyterian. Each community is a relatively recent settlement (all were founded by mission stations during the late 19th and 20th centuries); has a resident VCC fieldworker; and belongs to a different vernacular language group.

Although the communities have different social and cultural characteristics, all interviews are treated as part of the same sample population. The cultural diversity of Malekula and Vanuatu means that our ability to generalise from case study data is limited; however, the issues faced by the focus communities and described herein are likely to be typical of issues facing rural populations throughout the country.

### Field methods

Data were gathered using semi-structured interviews, which offer the flexibility to gather a wide range of information, and allow the interviewer to gain a more nuanced understanding than a structured questionnaire [73]. We identified three key groups of stakeholders (Table 1): locally identified TEK experts and schoolteachers within the focus communities, and officials (including policy makers, VCC staff, and academics) in the capital Port Vila. These stakeholders were identified as being directly affected by any moves to integrate TEK and formal education or as being involved in forming education policy.

**Table 1:** Interviewee characteristics for semi-structured interviews

		TEK experts	Teachers	Officials
	Total *n*	27	12	10

**Gender**	Male	21	10	6
	Female	6	2	4

**Place**	Wintua	4	4	0
	Dixon Reef	10	2	0
	Tisvel	8	3	0
	Unmet	5	3	0
	Port Vila	0	0	10

**Position**	Chief	11	0	0
	Fieldworker	4	0	0
	Primary teacher	0	7	0
	Secondary teacher	0	5	0
	Academic	0	0	5
	VCC staff	0	0	3
	Education officials	0	0	2

**Age cohort**	18-30 years	3	1	0
	31-60 years	14	6	5
	60+ years	10	5	5

Interviewee selection was purposive and non-random, and contacts were gained through snowball sampling methods [73]. For TEK experts and schoolteachers, our initial participants were selected through consultation with the VCC fieldworker in each community, and additional participants identified through recommendations of previous interviewees. In Port Vila, initial contacts were made through literature searches and key contacts at the VCC, and subsequent contacts through recommendations of previous interviewees. We recognise the bias inherent in snowball sampling, as the contacts one gains are dependent on the social networks of the gatekeeper informants [73].

The researcher lived in each of the four communities for one to two months between 2008 and 2010 and conducted interviews over this period. Interviews were conducted as part of a larger research program and covered a number of topics. The interview schedule was similar for each stakeholder group, but differed in the specific questions asked. For community-based interviews with TEK experts and teachers, the interview focussed around the magnitude and nature of changes in TEK and TEK transmission within the community; perceptions of the principal drivers of these changes; perceptions of the effects that these changes have had within the community; the ability of TEK to interact with the formal school system; and perceptions of specific areas of synergy. For interviews with Port Vila-based officials, questions focussed on the drivers of TEK and linguistic erosion; specific issues around curriculum and policy design; and barriers to the integration of local and formal education systems. Questions were piloted with VCC staff or fieldworkers to ensure salience. All interviews followed a list of key questions, but our approach was flexible to enable us to follow up leads and stories where pertinent.

All interviews were conducted by JM in Bislama or English according to the preferences of the interviewee. Interviews were conducted strictly according to a code of ethics adapted from the International Society of Ethnobiology [74], and approved by the Human Ethics Committee at Victoria University of Wellington (approval number 16500) and the Vanuatu National Cultural Council under their Cultural Research Permit program. All participants were adult and gave their full, prior, informed verbal consent to the interview process. Participant identities remain confidential.

### Methodological limitations

There were four key limitations to the methods outlined above. First, the use of in-depth qualitative methodology limits the sample size and thus constrains direct comparison with other studies or between groups within our data (for example, between the focus communities). Second, our sample selection was limited and excluded other relevant stakeholders (such as pupils and regional education officials) for ethical and logistical reasons. Third, the interview schedule did not identify specific mechanisms to include TEK in the school system.

Fourth, and critically, samples of TEK experts and teachers within the communities displayed significant gender biases. For the teachers grouping this may have been because there were more male teachers than female in the general population. For the TEK experts grouping, however, the bias resulted from methodological constraints, based on two primary factors. First, our initial interview targets (VCC fieldworkers) were all male, and tended to recommend men as experts for future interviews. Second, we found that women were less comfortable with the interview process and would often decline to be interviewed, possibly due to the presence of the researcher (a male New Zealander).

### Analysis

Interviews were analysed using thematic coding based on an inductive approach. Coding was based on the approaches outlined by Miles and Huberman [75] and Bernard [73], and was completed in several distinct stages.

In the first iteration, we read all of the transcripts in order to gain a basic understanding of the responses. During this stage, we made a list of initial codes in the margins of the transcript, and used these labels to develop a general category scheme of participant responses. Second, we began to identify themes by sorting the initial scheme into concrete categories and subcategories. This categorisation reflected the frequency of response as well as the similarity between interviewees. Third, we re-read the transcripts to identify atypical and dissenting cases. The themes developed during this section form the paragraphs of the results section below. Last, we reviewed the themes and evaluated their relationship to the literature.

## Results

Results are presented here according to the key questions set out at the end of the background section.

### Could TEK be legitimately included into the formal school system?

All participants noted that TEK in Vanuatu had eroded over recent generations. This corroborates other data from the same research program, which (using a structured interview) indicated that 96% of 120 participants around Malekula perceived TEK to be eroding (McCarter, unpublished data). Thirty of the 49 interviewees noted that the formal education system had played a key role in the erosion of this knowledge, alongside other key drivers such as church influence and community inattention. Participants noted that formal education drives TEK erosion through introducing new, competing, forms of knowledge; through promoting the use of English, French and Bislama over vernacular languages; and through a lack of integration with the wider community. As expressed by one elder at Unmet: *"The kids go to school, and they catch some thinking that isn't really good - they learn knowledge, but they do not learn wisdom" *(Male, 62, TEK expert).

**Figure 2:**
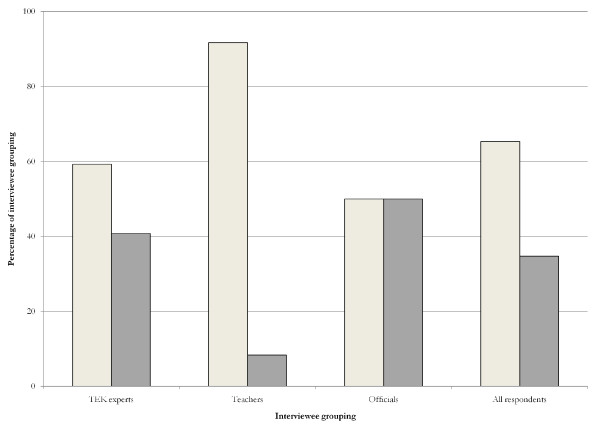
**Percentage of respondents per grouping who support the teaching of TEK in formal school**. Light bars indicate assent, dark bars indicate dissent.

Although 65% of respondents believed that TEK could be included in the formal school system, we found clear differences in the responses from different interviewee groupings (Figure 2). Teachers and community TEK experts were more likely than not to agree that TEK could be legitimately included in the formal education system.

Participants who believed that TEK could be included in formal school systems gave a number of justifications, which coalesce into two main themes. The first group (n = 15) were not concerned about the negative consequences of such a move, and noted that TEK would be a valuable means of counterbalancing the western knowledge that is currently promoted by the school system. For example, one official described how TEK could enhance educational outcomes: "*What I'm saying is there have been some things that have been learnt over centuries, over thousands and thousands of years, that have worked...and these are basically to do with interaction of people, and how they interwove *[sic] *all the different aspects of a community together to work together to achieve what we want today - and that's basically peace" *(Male, c.60, Official). A teacher also stressed the role that TEK could play in education: "*There are now two roads on offer *[western and *kastom*], *and it is hard for them to choose. People need their culture and traditions to be taught to them, to give them roots and make sure this choice is easier*" (Female, c.40, Teacher).

The second group (n = 9) were pragmatic and were concerned with exploiting some of the characteristics of the formal education system to increase TEK transmission. The formal school aggregates pupils, and thus presents the opportunity of a captive audience for TEK transmission. As noted by an elder at Wintua: *"...now that things are like this, it is too hard to call the kids to one place. They go this way and that all the time - but when that school bell rings, everyone goes there. That's why we need to teach customary knowledge at school" *(Male, 65, TEK expert). Moreover, formal education represents a large time commitment, which participants perceived could profitably be used for TEK instruction.

Figure 2 also indicates that a significant proportion of respondents (including 50% of officials) did not agree with the sentiments outlined above. A wide variety of reasons were given, which differed between the interviewee groupings; however in general, these participants were concerned that such a move would remove value, either from TEK itself or from the formal education experience.

TEK experts were concerned that integrating TEK and school might devalue the knowledge. Three key reasons were given: first, by inadvertently increasing the transmission of negative elements of *kastom*, including witchcraft and sorcery [cf. [76]]; second, by facilitating the teaching of gendered and secret aspects of TEK to a wider audience than would otherwise be intended; or third, by promoting the teaching of TEK by the wrong people, if appropriate teachers were unavailable. These participants noted that the transmission of TEK is inherently contextual, complex, and difficult to generalise into the school environment. This was wryly noted by one elder: *"..*.[take] *this leaf, for example, one of its medicines you can teach in public, but the other way of using it you definitely cannot. It is secret*" (Male, 66, TEK expert). Moreover, there was also some concern that teaching TEK in a classroom, an essentially passive form of knowledge transmission, would disconnect the TEK from its practical reality. This viewpoint was supported by one official: "*...the system as it is puts the kids in an artificial world that does not belong to them. But putting TEK into the school curriculum would force them to passively pick up the knowledge, rather than actively. This would change the knowledge...and surely that defeats the purpose of putting it in school?*" (Female, c. 35, Official)

Other interviewees, in particular officials and teachers, focussed more on the impact that the introduction of TEK would have on the educational environment. There were two key concerns: the first, shared by two TEK experts as well as officials and teachers, was that teaching TEK at school would dilute the educational experience for pupils, in particular by interrupting the teaching of English or French. This would then impact on the ability of pupils to attain employment in urban centres such as Port Vila. One secondary school teacher noted "*...it is part of the culture here that parents want their children to learn newer, better things that are different to what they know already. They want their children to be prepared for white-collar jobs...there are plenty who look down on the old ways as lower class*" (Male, c.40, Teacher). Other teachers considered that such initiatives would compete for time in an already packed schedule. For example, social science, which is one area where TEK might be integrated, was only allocated 1 hour and 15 minutes of the school week, as opposed to 17 hours for Maths and English combined (McCarter, unpublished field notes).

Interviewees also mentioned several structural and epistemological barriers to the teaching of TEK in school, which are expanded on below.

### How might TEK be integrated into the formal school system?

When asked about appropriate subject material for the teaching of TEK in school, interviewees (mostly TEK experts) identified six discrete domains of knowledge that would be suitable for inclusion as subjects in the formal school system (Table 2). The first three (medicine, agriculture, and construction) refer to theoretical and practical TEK skills that participants felt are at risk of not being effectively transmitted and would translate well to the school environment. That is, they could conceivably be taught in a classroom through instruction from local resource people. Participants noted that the inclusion of TEK such as this in curriculum modules would increase intergenerational transmission of these subject domains; would make the younger generation more 'useful' in the community environment; and would increase their self reliance.

**Table 2:** TEK subject domains for inclusion in the school curriculum

Subject domain	Featured skills	Support from TEK experts (n = 27)	Support from teachers (n = 12)	Support from officials (n = 10)
Traditional medicine	Plant naming, illness naming and recognition, medicinal preparation	9	0	0
Agriculture	Knowledge of traditional calendar, agricultural techniques, respect for appropriate social restrictions	7	3	0
Construction	Weaving bamboo walls, trying thatch with vines, construction of '*kastom*' style houses	7	1	0
Resource management	Recognition of appropriate ownership and restrictions on natural resources	5	2	2
Respect	Fostering of appropriate attitudes and values to property, people, and natural resources	6	0	0
Vernacular language	Linguistic skills, vernacular literacy, learning of *kastom *stories and histories	10	7	6

In recommending the next two subject domains (resource management and respect) participants were more concerned with ensuring the transmission of appropriate norms and values to ensure cohesion within the community environment. This was considered critical for the effective governance of natural resources (such as through spatial and temporal restrictions on resource use known as *tabu*) and to foster appropriate attitudes to persons and property within the community. Participants commented that the individualistic ethic of the formal school system had resulted in a lack of respect for traditional institutions, and had increased incidences of theft or lack of appropriate deference to social hierarchies. There was some dissent on whether norms and values could be transmitted in school, which we return to in describing the barriers below.

Vernacular language was the most commonly discussed subject domain for potential inclusion in the school curriculum. The debate over the place of vernacular languages in the national curriculum in Vanuatu has continued for some years, and while some faltering steps have been made, there has been little real progress on the ground [77-79]. Participants often noted that education in vernacular languages also implicitly teaches medicinal plant knowledge, social titles, agricultural techniques and the myriad other facets of TEK that are embodied in any particular language. Moreover, proficiency in a vernacular language fosters a sense of connection to place and land. This is true in both an esoteric and practical sense: if a person is not able to describe the boundaries of their family land in the vernacular, they risk losing much of their legitimate claim to that land. This is a worrying development in a time when customary title is often under dispute [80].

TEK experts noted that there would need to be changes to the current system for the appropriate inclusion of the subject domains from Table 2 in formal education. First, there would need to be systematic facilitation of access for local experts to the school system. School teachers are commonly non-local and are untrained in TEK and *kastom*, and there are many areas within the subject domains that would need to be taught by community-selected experts. Bringing resource men and women into the school system may also need to be supported by some form of cash payment. Second, the community experts would need to have some degree of autonomy over which aspects of TEK were taught at what time. This was mainly because, as noted above, there were concerns about negative aspects of TEK that would need to be tightly controlled from within the community. Third, there would need to be regular time created within the current curriculum setup to allow the effective transmission of TEK.

Several schoolteachers and officials (n = 7) added a qualification to their support for TEK in formal education by noting that the timing of such a move would be critical. In particular, they noted that vernacular language and/or TEK could be appropriately included in school at the primary level only. If included from an early age, participants noted that this would provide a 'base' upon which the other educational necessities could be built. Respondents felt that by the time pupils reached secondary school it was too late as the pupils were too old to acquire TEK and language effectively, and the time available in the curriculum is too limited. A senior teacher summed this position up: *"...we should teach vernacular, but only at the lower levels. The kids come here, and the parents pay for them to be here, to learn English. We don't want to hold them back. At this stage, they need to gather the literacy and numeracy skills that will help them in the future" *(Male, c.50, Teacher).

One official (Male, c.60) in Port Vila provided an instructive example of how TEK and formal school might be integrated from another island of Vanuatu (Futuna). For several years the interviewee has been working with the school board to break down the barriers between school and the community. They have been allowing regular slots within the school program for local experts to come and teach TEK, and have ensured community participation in the school board and regular contact with school authorities. The interviewee stressed that the focus has been on teaching values rather than specific skills. For example, he observed that being able to weave a mat is not the end goal of the teaching process, but rather the development of the patience and commitment to be able to complete the task. The interviewee noted that the program has been successful: as well as increasing intergenerational transmission of TEK, the school had also achieved one of the highest exam pass rates in Vanuatu. The philosophy of the approach is summed up in this excerpt: *"...basically, we are going back to the simple things like *[acknowledging] *that the school is a small part of the community. It's not the other way around, the community is not a small part of the school. The school is a small part of the community, that means that the school fits in to the local environment, it fits into the local chiefs, it fits into how they do things."*

### What are the possible barriers to teaching TEK in schools?

Interviewees highlighted multiple barriers to the inclusion of TEK in formal education on Malekula, of which several have already been noted. These can be broadly divided into practical and epistemological barriers (Table 3).

**Table 3:** Barriers to the inclusion of TEK in school on Malekula

Barriers to the inclusion of TEK in school	Support from TEK experts (n = 27)	Support from teacher (n = 12)	Support from officials (n = 10)
**Practical barriers**			
*Characteristics of formal school model*			
Inexpert teachers	12	5	2
Lack of time in curriculum	5	10	6
Lack of political support	4	6	7
*Characteristics of TEK*			
Increasing transmission of 'bad' *kastom*	9	0	2
Payment and availability of resource people	10	0	0
Lack of community support/ability	11	4	1
Linguistic/cultural diversity	3	7	4
**Epistemological barriers**			
Mode of knowledge transmission	9	5	6
Transmission of underlying values	4	4	4
Different knowledge systems	6	2	3

### Practical barriers

Practical barriers were associated both with characteristics of the formal school system and with the nature of TEK. Within the former grouping, issues with ensuring that the correct resource people are able to teach and that there was sufficient time in the curriculum have already been mentioned. An additional key dynamic is the lack of consistent political support for TEK and for vernacular language education. This was perceived to be a problem by the majority of officials and teachers. Vanuatu's political landscape is highly fractured, and governments and ministers change regularly. There are splits between Anglophone and Francophone sections of government, and if policy changes are enacted they often lack the resources to be implemented fully. Moreover, interviewees believed that until recently donor priorities (especially those of New Zealand and Australian aid agencies) had steered educational policy away from contextualised, indigenised, ni-Vanuatu education.

The sheer cultural diversity of Malekula was also cited as a barrier to the effective integration of TEK into the formal school system. This is particularly true in the secondary school setting, where students commonly come from around Vanuatu, and where multiple vernacular language groups are represented in a single classroom. Even in primary education, (where only one language group is usually represented) there may be many clan groups present, which is an artefact of the resettlement patterns that followed mission establishment around the island. Three of the TEK experts expressed concern that political allegiances would mean some families would be marginalised, or one dominant language would be privileged over others. As one of these TEK experts at Dixon Reef, where one vernacular language (Novol) is dominant, noted: "...*there are many different languages here, and if they do not stand strong, then Novol will come on top of each of them*" (Male, c.60, TEK Expert).

### Epistemological barriers

Interviewees also cited a number of epistemological barriers to the inclusion of TEK in the school system. Some of the most commonly expressed reservations were associated with differing methods of knowledge transmission between the formal school system and a traditional system. Formal school is based around a teacher-centred model in which one or two instructors dispense public knowledge to many learners, regardless of clan affiliation, gender, or age. This model was considered to be at odds with systems of TEK by several interviewees, who noted that much TEK was private and was held by particular family groups or individuals. Moreover, traditional routes of knowledge transmission would have been based around vertical (parent-child) means rather than horizontal (within peer groups) or oblique (one instructor to many learners) means. By way of example, in traditional medicine some use domains (such as that of how to treat common ailments such as headaches) are considered common property and could be taught at school. However, treatment of more serious illnesses or those with traditional aetiologies are the domain of certain individuals who have earned the right to use them from the previous holder of that knowledge.

More broadly, then, there was a common perception that while the Western-derived formal education system is based in an ethic of every student having the right to know everything, this is not always congruent with customary systems of knowledge transmission and acquisition. Moreover, practice and ownership of TEK was considered to be bound by an ethic of respect and by social norms that existed outside the transmission of the knowledge itself. The following quotation demonstrates that several interviewees considered the school system to be a limited conduit for the transmission of such values: "*I mean, if we are going to think we can teach these values by someone who is in the community coming to talk to them, basically what they can only teach are the things that you can see outside. But the real thing that should drive everything else...you cannot teach it like that. You teach by way of life...and if we don't do that, then it won't work..*." (Male, c.60, Official)

Underlying such concerns is a common conception that the two systems of knowledge transmission are fundamentally different. Ideally TEK is embedded in everyday life, whereas formal school is perceived as a discrete entity that exists outside the framework of the village. Whilst the transmission of TEK from teacher to learner is based in the practical reality of the lived environment, knowledge in school is theoretical, conceived and stored in paper and books. As such, those interviewees who did not support the teaching of TEK in school often noted that it was impossible to teach TEK adequately in the academic, formal context, as this would remove the connection between knowledge the teaching of practical skills. This fundamental disconnect was expressed by a female TEK expert: "...*in the time before, we didn't need to go to school to learn traditional knowledge - it was just life..." *(Female, 30, TEK Expert).

## Discussion

Interviewees outlined a number of key areas of synergy between the formal school system and TEK. Moreover, they noted that the flexibility to incorporate distinct domains of TEK could add value to the school curriculum and contribute to the maintenance of *kastom*. As such, our data corroborate the key arguments raised in the background section regarding the potential of TEK to contribute to formal education systems. However, the epistemological and practical barriers to teaching TEK at school suggest that there is reason for caution. In particular, interviewees noted the potential for adverse affects on both traditional knowledge and the outcomes of formal education.

The majority of interviewees noted that the formal school system does not fully meet contemporary needs or sustain traditional culture and TEK. This supports assertions made by some ni-Vanuatu writers, who contend that the education sector should be contextualised through the inclusion of local and indigenous content [e.g. [64-66], [81-83]]. Whilst the inclusion and support of traditional culture and TEK is only one aspect of the education system that needs updating, Vanuatu's formal school system has been described as an 'alienation agent' that has a tendency to remove children from the context of their traditional culture at an early age [66]. Moreover, comments during the interview process indicate support for the idea that Vanuatu schools may perpetuate a 'psychology of dependency' by de-valuing local knowledge relative to western knowledge and modes of learning [64].

Our findings also support the assertion that, *prima facie*, TEK could add value to the school curriculum in Vanuatu. This is no particular surprise, given that TEK has been shown to have immense value across a number of spheres over the past decades [84]. However, the value of TEK in this regard is critical, as countries across the Pacific region (including Vanuatu) are currently debating and enacting curricular reform [32-34].

Crucially, we note there are specific synergies between the various ways in which interviewees suggested that TEK might complement the school curriculum and the model of UNESCO-mandated reform proposed in the Delors Report [31]. This report suggested education policy should be reorganised around four 'pillars': learning to know, learning to do, learning to live together, and learning to be. The western model of education tends to focus on the first two pillars. However, the Delors Report accorded equal importance to latter two and emphasised the need for students to be aware of their values and place in the world. This report has had a significant influence on the direction of curricular reform in the region [33,35], and if TEK adds value to curricula by contributing to the four pillars it would strengthen the case for its inclusion in formal education systems.

The first two pillars ('learning to know' and 'learning to do') are concerned with cultivating a desire and ability to learn, and the skills to reflect learned knowledge with innate competencies. These two pillars have been the focus of education, both formal and informal, over the past decades in Vanuatu and the Pacific more broadly [85]. However in the Pacific context scholars have heavily criticised the status quo, noting that the knowledge and skills taught in formal school systems around the region are not representative of the depth and diversity of local knowledge [86,87]. Moreover, these scholars contend that conventional school curricula in the Pacific region are rooted in a foreign worldview that fails to build on existing competencies and impedes the transmission of traditional knowledge [69].

Interviewees noted specific domains of knowledge and skills (such as traditional medicine) that form discrete areas in which TEK could contribute to the school system. The identification of these focal areas suggests that the introduction of TEK into education might be a key step in localising the content and process of curricular delivery, thus strengthening the first two pillars as suggested in the Delors Report. Any such move would be supported by recent work which has documented distinct benefits of contextualising formal education using local knowledge [4,19]. Moreover, the maintenance and revitalisation of TEK knowledge and skills may have a direct impact on the adaptive capacity of the communities in question: for example, traditional methods of house construction have been shown to be a critical element of resilience to extreme weather events in other areas of Vanuatu [56,57].

However, TEK may be able to add value most significantly to the latter two pillars ('learning to live together' and 'learning to be'). The strengthening of these two pillars provides a particular challenge to curricular reform, as it necessitates a shift away from teacher-centred, exam-based learning, and may also require community support and participation [85]. The inclusion of these pillars in the Delors Report is an acknowledgement that education should consist of more than the passing of decontextualised knowledge and skills, and should contribute to the formation of identity in the individual and the eventual development of wisdom. These two pillars cannot be attached as discrete elements in the curriculum, and must be woven throughout each subject area [31,85].

We contend that the inclusion of TEK (especially the participant-identified domains of 'respect', traditional resource management institutions, and vernacular language) has potential to strengthen significantly the ability of education to address the latter two pillars. As several interviewees noted, the institutions that surround TEK are the key means of maintaining order and governing natural resources in these communities. The recognition and incorporation of these institutions, including the appropriate respect and acknowledgement of leaders, into the school curriculum might be a key way of ensuring that 'learning to live together' is adapted to the village environment. In turn, this would assist in the promotion of resource management for resilience, and may foster the understanding of ecological processes [88]. Moreover, the integration of vernacular language and TEK teaching in the formal school system would ensure that students developed a sense of membership of their particular clan, village and island [66].

### Barriers to including TEK in the formal education system

Although there is potential for TEK to add value to the existing school curriculum in Vanuatu, its introduction may be constrained by multiple challenges. Interviewees noted three key tensions inherent in the geographic and social context of TEK:

#### 1. Diversity vs. centralisation

TEK, as a localised entity, is unlikely to be widely applicable outside the environmental and social context in which it has evolved [89]. Therefore, school curricula that involve TEK must be flexible enough to incorporate local views and empower TEK holders, despite emanating from a central government. This may involve the use of specific place-based assessments [cf. [9]], but this process would have to be thorough and carefully managed in a culturally diverse nation such as Vanuatu

#### 2. Public vs. private knowledge

Intellectual property becomes a key concern with any attempt to formally include TEK in 'public' education. TEK is often intimately connected with social order and family groupings, and may not be appropriately shared with the wider community. Therefore, community heterogeneity must be taken into account [90]. This also indicates that the inclusion of TEK in formal education is likely to be only one of a range of tools needed for the maintenance and revitalisation of traditional knowledge.

#### 3. Vertical vs. horizontal knowledge transmission

In other areas of the world, cultural transmission of TEK has been shown to be predominantly vertical (parent to child) [e.g. [21,22]], and this is likely to be the case on Malekula. Integrating TEK into formal school may shift the mode of knowledge transmission from vertical to horizontal (within peer groups) or oblique (one instructor from the parental generation to many younger learners). This may result in a fundamental change in the structure and content of TEK, because the type of transmission pathway can influence the characteristics of that body of knowledge. For example, while vertical transmission results in slow rates of adaptation, horizontal and oblique methods can result in rapid diffusion and spread of new ideas [21,91]. A crucial facet of this tension is the shift from oral to written forms of knowledge transmission.

Perhaps more importantly, however, these findings draw attention to significant epistemological barriers to the integration of TEK and the formal school curriculum. Although the boundaries between indigenous knowledge and western knowledge have been argued to be largely arbitrary and unhelpful [89], it is clear that interviewees considered there are fundamental differences in the two systems of knowledge transmission on Malekula. This is corroborated by research indicating that TEK transmission is usually experience-based, learner-centred, and acquired through social interactions such as play, in contrast to knowledge transmission in the formal school system [23-25].

Within the Pacific, other research has found that imported education systems are, indeed, 'antithetical' to local means of indigenous education [87]. Such work has argued that irrespective of the content of the curriculum, the makeup and structure of the school mean that it transmits essentially foreign values [85-87]. These values are transmitted through the ethic of the learning environment, as Sundar [42] notes in the global context: "...critical education theorists have long since laid to rest the idea that curricula involve an innocent transmission of 'knowledge' that is not at the same time inflected by race, class, or gendered assumptions, or that pedagogy does not involve moral projects of transformation" (p 374). As such, it would be extremely challenging to teach TEK in a western setting in a way that would not emphasise those foreign values, which may in the process invalidate the TEK [6,92]. This was reflected in the comments from interviewees who were concerned that the inclusion of TEK in the school curriculum might implicitly erode traditional means of transmission.

Underlying all the barriers are issues concerning the power and sovereignty of local and indigenous peoples over the education of their younger generations. The validation and incorporation of knowledge in the formal school system is an immensely powerful act, as this knowledge has a claim to 'truth' that others do not [37]. The holders of TEK should be in control of this process, as actors in positions of power (such as academics or policy-makers) can influence the choices that local people make about what is desirable and valuable in their own paradigms [93]. This will, in turn, require significant attention to the ways in which curricula are developed and implemented, as "...without explicit and continuing attention to how power structures knowledge, it will remain impossible to achieve the aim of working in the interests of indigenous or other marginal peoples" [94: p 295]. As such, the conservation of TEK and the strengthening of education curricula should avoid a focus on specific pieces of knowledge in isolation from their cultural context. A more appropriate focus may be on sustaining the institutions and worldviews in which that knowledge is embedded [94].

### Moving forward with TEK maintenance and revitalisation

There are, then, significant issues that might impact on the value that formal education systems might have to TEK maintenance and revitalisation. We argue, however, that approaches that facilitate a high level of local participation in teaching and unit design may offer profitable future pathways for TEK maintenance.

One such approach, implemented in one school on the island of Futuna, was noted in the results section above. Another example is the VCC's recent design of a series of units for years one to eight, which aim to involve community members and incorporate TEK into the science curriculum [71,95,96]. These units do not seek to document specific details of TEK within the curriculum, but rather focus on encouraging pupils to seek out experts in their communities and discuss various aspects of the natural world. They also contain a dedicated teacher-training component to ensure that teachers have the skills to facilitate increased contact with the community. At the time of writing, the units were being distributed to regional education officials around the country and were intended for use in the 2011 year. No information is available at the present time as to their success or otherwise, however these units represent a promising and innovative means of increasing intergenerational TEK transmission.

Because formal school is unlikely to be able to maintain all aspects of TEK, other means may also be necessary. Of the other available means for TEK maintenance, *in situ *revitalisation efforts appear to hold the most promise [97]. Globally, *in situ *TEK maintenance initiatives have been created with a wide range of objectives, including the promotion of vernacular language, campaigns for human and land rights, and increasing the consumption of traditional foods [97]. Other approaches seek to meld biological and cultural conservation goals for integrated biocultural conservation [see [98]]. On Malekula, a series of local '*kastom *schools' (small local organisations, independent of the formal school, for the teaching of *kastom *and TEK) provide an interesting case study of *in situ *TEK conservation (McCarter and Gavin, in preparation). In general, approaches that address the fundamental issues of power imbalance, control over intellectual property, and TEK erosion have promise. However, as noted, there is a real need for more research attention to the challenges inherent in maintaining and revitalising TEK, vernacular language, and cultural continuity more broadly.

## Conclusions

We find that TEK may be able to add value to the formal school system in Vanuatu, especially with regards to curriculum reform via the model set out in the Delors Report. In addition, this may assist in the maintenance of ethnobiological knowledge. However, we note that the value of formal education to TEK is less assured, and that overcoming the practical and epistemological barriers outlined above will require considerable effort. Indeed, to do so may require a substantial redesign of the entire school system, to allow for not just the dissemination of other forms of knowledge but also to empower other ways of being, knowing, and learning. However, critically, we note that the desire for such a radical change in education policy may not always exist at the local level. Ideally then, local people would have more power to determine the content and structure of the education system.

Education reformists, ethnobiologists, and practitioners of cultural conservation have all called for the inclusion of TEK in the formal school system. However, little research has occurred examining the feasibility of this approach. What is now needed is more detailed research on how to cope with the kinds of barriers identified here, or to determine if other modes of TEK conservation would be more practical. As a discipline, ethnobiology is in a unique position to assist the conservation of biocultural diversity, and a more systematic examination of the potential options for the maintenance and revitalisation of TEK will be a vital contribution over the coming years.

## List of abbreviations used

TEK: Traditional Ecological Knowledge; VCC: Vanuatu Cultural Centre.

## Competing interests

The authors declare that they have no competing interests.

## Authors' contributions

JM designed research, collected and analysed data, and drafted manuscript; MCG participated in study design, helped to draft and edited the manuscript. Both authors read and approved the final manuscript.

## Endnotes

^i ^We define TEK following Berkes [84] as a "...cumulative body of knowledge, belief and practice, evolving by adaptive processes and handed down through generations by cultural transmission, about the relationship of living beings (including humans) with one another and with their environment" (p 7). As such, it represents the subset of traditional knowledge that is concerned with the environment, and is the manifestation of centuries of human-nature interaction [99].

^ii ^For our purposes, this broad definition will suffice. In reality, *kastom *is complex term with considerable political and historical weight [64,100]. In a more complete definition, Bolton [101] notes that: "*'Kastom' *is a cognate terms for culture in Bislama...it is used to refer to knowledge and practice that ni-Vanuatu understand to be authentically their own, deriving from their pre-colonial past and from their place...it is a term that derives from contact with outsiders yet describes what belongs to people of the place "(p 6).

^iii ^At the time of writing (August 2011) there are reports that the Vanuatu Ministry of Education has recently instituted vernacular education in schools between kindergarten and year three. At this stage, we are unaware of published accounts of this program.

^iv ^A semi-autonomous public institution charged with the maintenance and revitalisation of ni-Vanuatu culture, under the direction of the Vanuatu National Cultural Council.
